# West Chicago Medical Society

**Published:** 1880-05

**Authors:** 


### ARTICLE IX.

### WEST CHICAGO MEDICAL SOCIETY, February 9, 1880.

### DR. E. W. LEE ON TETANUS.

Some five weeks ago I was called to see a man who, seven weeks before, through careless driving, was thrown from his wagon and dragged some 75 to 100 feet, crushing the lower part of the leg.

A doctor was called, who said it was only a flesh wound. He did it up with some simple dressing and the man was carried home. About a week later the man noticed that his leg was not getting better, so he sent for another doctor.

The second doctor examined it, and thought it would be healed in a few days. Four or five weeks after, he sent for me.

I noticed the man had a peculiar expression of countenance—that risus sardonicus, that puritanic grin that marks a case of tetanus. It was by mere accident that I noticed it. He complained of tightness of the chest, as though a belt was fastened around him. He had had this symptom nearly ever since the injury. I thought, from examination, that the tetanus had assumed a chronic form, especially from the length of time elapsed.

I put him on bromide of potassium and large doses of hydr. chloral, giving (grs. xx–xxx) 1.3 to 2 grams to keep him in stupor all of the time. The next day he was worse. I thought best to make an examination. Did so, and found, on making an incision, probably (1 in. to 1½ in.) 2½ to 3½ cm. of the fibula was laid bare, and found to be in a necrosed condition. A regular section of the fibula was made and the necrosed portion taken away. The wound was dressed under spray. His wife rendered the dressing futile by dressing with water. Then I dressed it with carbolized water, and afterwards with carbolized cosmoline.

The case went on getting worse. In just one week he had a case of acute tetanus. He had convulsions, and the muscles of the abdomen were in a state of tonic spasm. All of the muscles were in a state of spasm. The pulse began to rise. The temperature did not rise.

I sent for calabar bean. Commenced by giving (grs. $\frac{1}{8}$–$\frac{1}{4}$) 0.01 to 0.02 centigrams every two hours by the mouth, without the slightest effect.

Dr. —— was kind enough to attend the man during the evening ; then sent me word that he could not stay during the night, so I telephoned Dr. Murphy at the hospital, and he came down and stayed with the man during the night. I sent and got Squibb's calabar bean, and gave four injections, under the skin, of (gr. $\frac{1}{8}$) 0.01 centigrams, the first hour. The convulsions abated in frequency. By the time Dr. Murphy left in the morning, it was only necessary to give the injection once in two hours.

With each day there was progression after administering under the skin. If the remedy was not given as frequently, then the convulsions would come on and gradually resume their violence. The drug had controlling influence on these convulsions. By the seventh day an injection in the morning controlled it all day long. The improvement was steady and progressive as long as the drug was given. The man has made good recovery. He has not had any symptoms for seven weeks.

DR. HOLMES related a case of his where the tetanus commenced on the eleventh day after the injury or fracture of a finger. He commenced by giving (grs. ss–j) 0.03 to 0.06 grams every three hours (Squibb's). "To control the pain I gave a little morphine. Did not give enough to affect the pupils of the eyes. I attributed the effect on the eyes to the calabar bean. I gave (grs. ss) 0.03 gram doses by the mouth every three hours, without variation, from the time of the first symptom of tetanus to the conclusion of the disease."

Dr. Harcourt: A week ago to-day I was called to see a young girl, sixteen years old, who had fallen over a pail with a lamp in each hand, suffering a severe cut and wounding the thumb across the hand (right hand) down to the little finger, cutting a portion of the deep palmar arch, cutting with the glass a wide and gaping wound. It was two hours after the accident before I saw her. A neighboring physician had first come in response to a telephonic call. We cleansed the wound as well as we could, put in a number of stitches, fastening very closely together in that position.

In thinking of a case recently reported, it came vividly to mind that this would be a good case for tetanus, for the public opinion of the laity and profession is that an injury near the thumb or great toe is very likely to result in tetanus, and because the wound was cut with glass. I put her on potassium bromide and morphia. She took, during the night, (grs. ij) 0.12 grams of morphia, and (℥ij) 60 grams of bromide of potassium. Did not secure any rest until four o'clock in the morning. Next day gave hydr. chloral (grs. x) 0.7 grams. Continued this until Saturday without any symptom of tetanus.

The second day the family physician was called. The case went on favorably until Saturday night, when I received a note saying that "we had called in our family physician," and thus ceased further attention in the case.

General questions were asked and answered, and discussion continued for some time.

Dr. Lee continued by relating his experience with a child which had fallen some ten or twelve feet, and fractured the skull some (5 in.) 12 cm. in length. By using the trephine, he relieved the child and made it conscious. The child died at last, evidently from the effects of septic poison.

Followed by remarks by Dr. Harcourt, Dr. Lyman and Dr. Seeley.

POISON BY CHLORAS POTASSII.

Dr. Adelia Barlow: I shall attempt to give only a brief outline of three cases, one occurring three years ago, another about a year ago, and the last six months ago. I was very certain that the symptoms were those of poison. The first case was scarlet fever; a child nineteen months of age. The child had been treated with iron, quinine and potassium. The quinine and iron were given every hour; chlor. potass. every two hours. The saturated solution of chlorat. potass. contained (grs. v) 0.3 grams of the chlor. potass. to (ʒj) 4 grams; therefore, about (grs. 60) 4 gms. were given in twenty-four hours.

The child improved under the treatment, and I supposed was out of danger. The mother disliked to give the quinine on account of the bitter taste, and gave it only three times during the day. I requested her to keep up the potassium, as I regarded it a certain specific to that poison (scarlet fever). I did not intend to see the child again that evening, but as I had been in the habit of doing so two or three times a day, I felt as though I would go and see the child, and did so, and found the temperature of the surface of the body increased, the extremities cold and breathing stertorous. There was every evidence of poison, and I put him in a warm bath, gave a large dose of quinine immediately, and ordered brandy. They gave large doses of brandy, until eventually the child came out from under this influence and finally recovered.

In casting about for the cause of this sudden change, I feel it was due to the chlorate of potassium. On investigation, I find in the literature on the subject there was only one case where anything of the kind had been reported. The dose was not unusually large. In the United States Dispensatory it is recommended to give as large doses, and even for a long time as high as (ʒiv) 16 grams in twenty-four hours.

The next case was a child about eight months old, as near as I can recollect. In this case it was given in very large doses, half teaspoonful of the saturated solution every three hours. In addition, I had them give brandy with this dose. It got about ten drops to each dose of about (grs. ijss) 0.16 grams of the chlorate of potassa. The child took two or three doses before the symptoms of poisoning occurred, and the same symptoms as in the first case.

The last case was an infant three weeks old, with a case of thrush. It only took one dose of the medicine. The mother saw the medicine was poisonous, and would not give any more.

The symptoms appeared in a very few minutes after the dose was administered.

Since that time I have felt rather afraid of giving chlorate of potassium. Although I had previously given it in the same doses as I had given in these cases, I suppose it might be idiosyncrasia.

DR. HARCOURT spoke of the poisonous nature of chlorate of potassium. He spoke of Dr. Fountain, of Iowa, falling a victim from taking a large dose—(℥ij) 60 grams. It reduces the action of the heart. A case was mentioned where an adult took (ʒv) 20 grams, and the pulse was reduced twenty beats per minute. Given in still larger doses, the heart's action might be completely stopped. The recovery under the use of brandy shows the action here on the heart.

DR. LYMAN stated that Dr. Fountain had experimented on the use of chlorate of potassium, and continued to increase the dose until death. It produces suppression of the urine. He also spoke of the effects on the protoplasm of the cell structures.

---

HYDROCELE TREATED BY INJECTION OF A SOLUTION OF PERCHLORIDE OF IRON.—M. Houzé de l'Aulnoit. (*Bull. de l'Acad. de Méd.*, Feb. 10, 1880, p. 134).

The following is a résumé of a paper read before the academy of medicine, February 10th:

Instead of using a solution of iodine, which, pushed with too much force, may flow back into the scrotal envelopes and cause their gangrene, the author sought to avoid this grave complication by a simple hypodermic injection of a solution of perchloride of iron. The author cites fourteen cases, of which one recurred and all the rest were cured without the slightest induration of the gland. The author uses two drops of perchloride of iron to a (gtts. xx) gram and a half of distilled water; the tunica vaginalis is completely emptied, (℥j) 30 grams of the serous fluid are allowed to flow back, and then the feeble solution of iron causes the desired coagulation.

